# Inhaled loxapine and intramuscular lorazepam in healthy volunteers: a randomized placebo‐controlled drug–drug interaction study

**DOI:** 10.1002/prp2.194

**Published:** 2015-12-17

**Authors:** Daniel A. Spyker, James V. Cassella, Randall R. Stoltz, Paul P. Yeung

**Affiliations:** ^1^ Alexza Pharmaceuticals, Inc.Mountain ViewCalifornia; ^2^ Covance Clinical Research UnitEvansvilleIndiana; ^3^ Teva PharmaceuticalsFrazerPennsylvania

**Keywords:** Bipolar I disorder, drug–drug interactions, inhaled loxapine, lorazepam, pharmacodynamics, schizophrenia

## Abstract

Pharmacodynamic effects and safety of single‐dose inhaled loxapine administered via the Staccato^®^ system and intramuscular (IM) lorazepam in combination versus each agent alone were compared in a randomized, double‐blind, crossover study in healthy volunteers. Subjects received: inhaled loxapine 10 mg + IM lorazepam 1 mg; inhaled loxapine 10 mg + IM placebo; IM lorazepam 1 mg + Staccato placebo in random order, each separated by a 3‐day washout. Primary endpoints were maximum effect (*minimum* value) and area under the curve (AUC) from baseline to 2 h post treatment for respirations/min and pulse oximetry. Least‐squares means (90% confidence interval [CI]) for concomitant treatment versus each agent alone were derived and equivalence (no difference) confirmed if the 90% CI was within 0.8–1.25. Blood pressure (BP), heart rate (HR), sedation (100‐mm visual analog scale), and adverse events (AEs) were recorded. All 18 subjects (mean age, 20.4 years; 61% male) completed the study. There was no difference between inhaled loxapine + IM lorazepam and either agent alone on respiration or pulse oximetery during the 12‐h postdose period, confirmed by 90% CIs for AUC and *C*_min_ ratios. BP and HR were no different for inhaled loxapine + IM lorazepam and each agent alone over a 12‐h postdose period. Although the central nervous system sedative effects were observed for each treatment in healthy volunteers, the effect was greater following concomitant lorazepam 1 mg IM + inhaled loxapine 10 mg administration. There were no deaths, serious AEs, premature discontinuations due to AEs, or treatment‐related AEs.

**Abbreviations**
AE: adverse event
AUC: area under the curve
CI: confidence interval
CNS: central nervous system
ECG: electrocardiogram
FDA: US Food and Drug Administration
IM: intramuscular
IV: intravenous
PD: pharmacodynamic
VAS: visual analog scale

## Introduction

Agitation is often encountered in medical settings and is one of the most common manifestations associated with psychiatric disorders such as schizophrenia and bipolar I disorder (Alderfer and Allen 2003; Marder 2006; Hankin et al. 2011). Current treatments for agitation comprise typical antipsychotics, administered either alone or with benzodiazepines, and atypical antipsychotics (Wilson et al. 2012). These are available as intramuscular (IM), intravenous (IV), and oral formulations. IM and IV formulations are fast acting but invasive, whereas oral formulations are noninvasive but have a slower onset of action (Hankin et al. 2011), which may allow symptom escalation. One of the main criteria for selecting medication for the management of acute agitation has been identified as the speed of onset of effect (Battaglia 2005; Citrome 2013).

Oral loxapine, which was first approved in 1975, is well established for the treatment of schizophrenia (Paprocki and Versiani 1977; Heel et al. 1978; Dubin and Weiss 1986; Chakrabarti et al. 2007). An inhaled formulation of loxapine, administered via the Staccato^®^ system (Adasuve^®^), which is approved in the United States, Europe Union, and Latin America for the treatment of acute agitation associated with schizophrenia and bipolar I disorder (FDA 2012; EMA 2013b), offers a noninvasive treatment option combined with a rapid onset of action, similar to that of IV‐administered antipsychotics (Spyker et al. 2010). Inhaled loxapine demonstrated efficacy in the treatment of agitation in phase 3 clinical trials in patients with schizophrenia (Lesem et al. 2011) and bipolar I disorder (Kwentus et al. 2012), significantly reducing agitation compared with placebo within 10 min after the first dose, and was also well tolerated in these patients (Allen et al. 2011; Currier and Walsh 2013).

In these phase 3 studies (Lesem et al. 2011; Kwentus et al. 2012), lorazepam use was permitted as a rescue medication. Lorazepam is one of the most common benzodiazepines used to manage acute agitation (Marder 2006) and is often concomitantly administered with antipsychotics (Currier et al. 2004; Demler et al. 2012). Several studies are available on the efficacy and tolerability of concomitant administration of lorazepam with antipsychotics, for example, haloperidol, olanzapine, and ziprasidone (Bieniek et al. 1998; Currier and Simpson 2001; Zacher and Roche‐Desilets 2005). However, as lorazepam is known to interact with drugs that act on the central nervous system (CNS) (Cobb et al. 1991; Zacher and Roche‐Desilets 2005), caution is warranted when used concomitantly.

To date, no studies have examined the potential interaction between lorazepam and inhaled loxapine. Therefore, this study was designed to assess the safety and pharmacodynamics (PDs) of concomitant inhaled loxapine and IM lorazepam administration in healthy volunteers compared with each drug alone.

## Materials and Methods

### Participating subjects

Eligible subjects for this study were healthy males or females aged 18–50 years, with a body mass index between 18 kg/m^2^ and 32 kg/m^2^. Subjects were excluded if they had a history of or presented with any cardiovascular disease or disorder; asthma; chronic obstructive lung disease; sleep apnea; psychiatric illness or mental disorder, except for short‐term situational anxiety or depression of <2 years’ duration; any substance abuse or addiction within the last 2 years; or if they used an inhaler prescribed for wheezing or bronchospasm. Females pregnant during the 6 months prior to the study were also excluded.

### Study design

This was a single‐center, randomized, double‐blind, double‐dummy, single‐dose, three‐period, three‐treatment, Williams square crossover, drug–drug interaction study preceded by an open‐label single‐dose treatment with lorazepam 1 mg IM + inhaled loxapine 10 mg in healthy volunteers to validate the dose selection and the dosing regimen (ClinicalTrials.gov NCT01877642). Subjects in the open‐label part of the study received a single dose of lorazepam 1 mg IM (West‐Ward Pharmaceuticals, Eatontown, NJ) + inhaled loxapine 10 mg and were not permitted to enroll in the double‐blind part of the study. Eligible subjects for the double‐blind study were randomized (1:1:1:1:1:1) to one of six treatment sequences, where they each received the following treatments in the order determined by their allocated sequence: single dose of lorazepam 1 mg IM and a single dose of Staccato placebo; single dose of lorazepam 1 mg IM and a single dose of inhaled loxapine 10 mg; single dose of placebo IM and a single dose of inhaled loxapine 10 mg. Each dose was followed by a washout period of ≥ 3 days. All dosing and treatment follow‐up in the study were conducted in a clinical research unit (Covance Clinical Research Unit, Evansville, IN). The subjects remained in the research unit throughout the study and were discharged 24 h after the last dose. Subjects were monitored for PD endpoints and safety.

This study was performed in accordance with the relevant US Food and Drug Administration (FDA) and EU guidelines, the International Conference on Harmonisation E6 Good Clinical Practice Consolidated Guidances, and the Declaration of Helsinki. Institutional review board approval was provided prior to the start of the study and all volunteers provided written informed consent.

### Compliance with design and statistical requirements

Randomization and blinding were not required for the open‐label dose selection part of the study. For the double‐blind crossover part of the study, group sizes were equal and the sample size and design were selected according to FDA guidance and the convention for this type of study (FDA 2001). A computer‐generated randomization schedule was used and blinding was performed by a pharmacist not involved in any other aspect of the study.

### Drug delivery system

**Figure 1:**
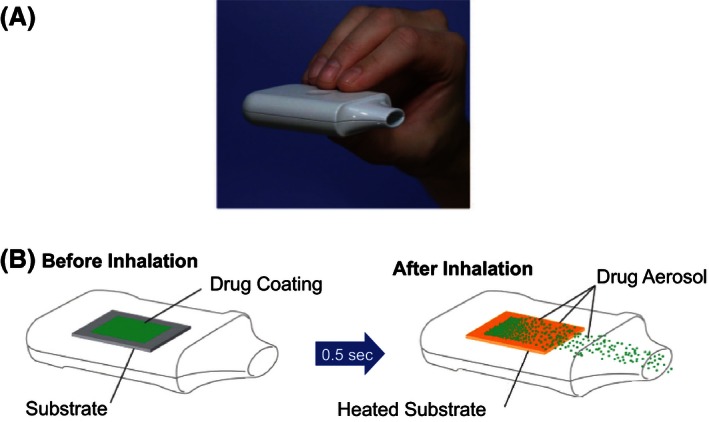
The Staccato delivery system (A) and schematic representation of the mechanism of drug delivery (B).

The Staccato system is a hand‐held drug product (Fig. 1) that facilitates rapid systemic delivery of loxapine via inhalation of a thermally generated aerosol (Dinh et al. 2011). A detailed description of the device has been presented elsewhere (Dinh et al. 2010, 2011). Briefly, oral inhalation through the product triggers the controlled rapid heating of a thin film of excipient‐free loxapine to form a pure‐drug vapor. The vapor condenses into aerosol particles within the device, with an appropriate particle size distribution for efficient delivery to the deep lung (Fig. 1) and provides an onset of clinical response within 10 min from administration.

### Assessments

#### Pharmacodynamics

The primary endpoints analyzed during this study were the maximum effect (i.e., *minimum* value) and area under the curve (AUC) from baseline to 2‐h posttreatment value in respirations/min and pulse oximetry between the treatment groups. The secondary PD variables measured were sitting blood pressure, heart rate, and sedation evaluated via self‐reported sleepiness with the 100‐mm visual analog scale (VAS). All PD variables were measured at various time points from 0–24 h post dose.

#### Safety

Safety was assessed by recording adverse events (AEs) after initial study drug administration and throughout the study, up to 30 days after the last study drug dose. Laboratory testing (blood chemistry, hematology, and urinalysis); vital signs (blood pressure, heart rate, respiratory rate, and temperature); 12‐lead electrocardiogram (ECG); and physical examinations were also performed at specified intervals pre and post treatment.

### Statistical analysis

Subjects who received at least one dose of the study drug in either the open‐label or the double‐blind part of the study were included in the safety population. The PD population included only subjects who completed all three double‐blind assigned treatment periods.

Least‐squares (LS) means and 90% confidence interval (CI) for the ratio of concomitant treatment versus either treatment alone were calculated for each maximum effect and AUC measure for each primary and secondary endpoint. The 90% CI for each ratio was compared with the log‐transformed range of 80–125% (the standard no‐effect boundary for bioequivalence). We concluded “no difference” when describing the results to mean that the combined treatment versus either treatment alone satisfied this criterion. All programming and analyses were performed using SAS software (version 9.2 or later; SAS Institute Inc., Cary, NC).

## Results

### Subjects

**Figure 2:**
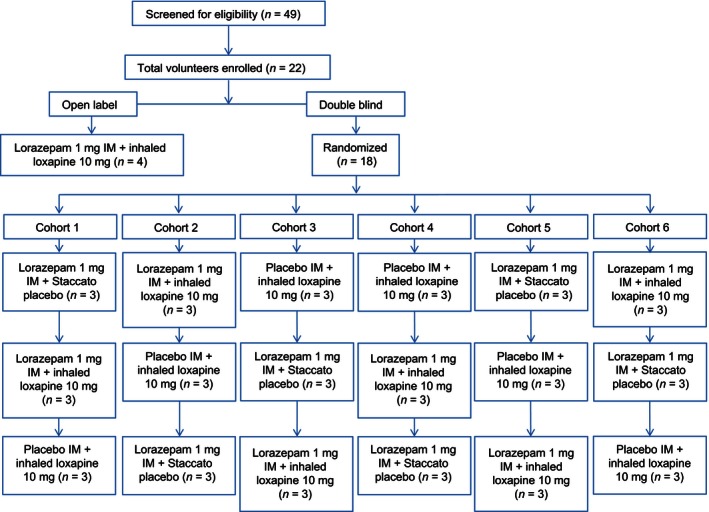
Patient disposition. IM, intramuscular.

Of the 49 screened subjects, four received open‐label treatment, and 18 were enrolled in the randomized, double‐blind, crossover part of the study (Fig. 2). All 18 subjects in the double‐blind part of the study completed all three treatments and were included in the PD population. All 22 participating subjects received at least one dose of study drug and were included in the safety population. The age (mean ± SD) of the safety population was 30.8 ± 9.3 years, most (59.1%) were male, and 13.6% were current smokers (Table 1).

**Table 1:** Demographics and baseline characteristics

Demographic or baseline characteristic	Open‐label part (*N* = 4)	Crossover part (*N* = 18)	Overall (*N* = 22)
Age (years)			
Mean (±SD)	32.5 (±9.68)	30.4 (±9.46)	30.8 (±9.30)
Median (min, max)	29.5 (25, 46)	27 (18, 49)	27 (18, 49)
Sex, *n* (%)			
Male	2 (50.0)	11 (61.1)	13 (59.1)
Race, *n* (%)			
Caucasian	3 (75.0)	10 (55.6)	13 (59.1)
Black	1 (25.0)	8 (44.4)	9 (40.9)
Weight (kg)			
Mean (±SD)	69.6 (±10.8)	73.1 (±69.2)	72.5 (13.7)
Median (min, max)	71.8 (55.6, 79.4)	69.2 (49.7, 96.4)	69.2 (49.7, 96.4)
Smoking history, *n* (%)			
Never smoked	2 (50.0)	11 (61.1)	13 (59.1)
Current smoker	1 (25.0)	2 (11.1)	3 (13.6)
Ex‐smoker	1 (25.0)	5 (27.8)	6 (27.3)

Max, maximum; min, minimum.

### Study drug dose

Combined lorazepam 1 mg IM + inhaled loxapine 10 mg produced a significant level of sedation in the open‐label part of the study in healthy volunteers; thus the maximum tolerated IM lorazepam dose was set at 1 mg and this was the dose chosen for the double‐blind part of the study.

### Pharmacodynamics

#### Respiration rate and pulse oximetry

**Figure 3:**
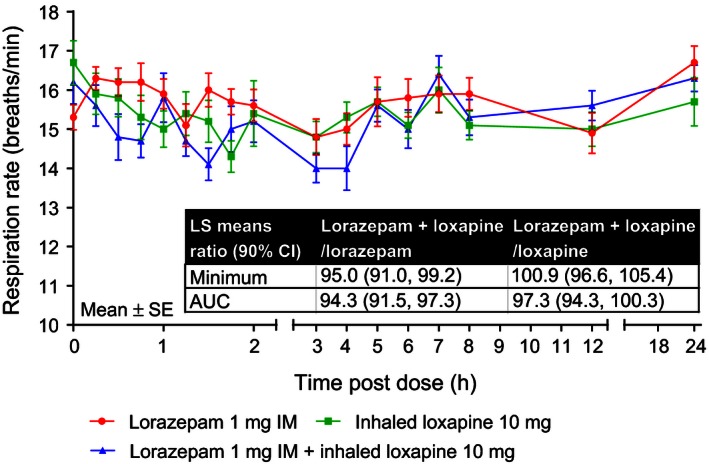
Mean respiration rate over time. AUC, area under the curve; CI, confidence interval; IM, intramuscular; LS, least squares.

**Figure 4:**
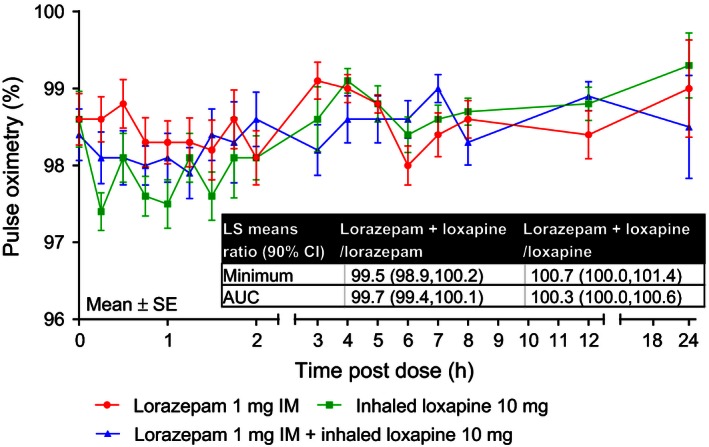
Mean pulse oximetry over time. AUC, area under the curve; CI, confidence interval; IM; intramuscular; LS, least squares.

There was no difference in the respiration rate minimum value or AUC (baseline to 2 h) with lorazepam 1 mg IM + inhaled loxapine 10 mg compared with either drug administered alone (Fig. 3). Similarly, no difference in the pulse oximetry minimum value or AUC was observed with lorazepam 1 mg IM + inhaled loxapine 10 mg compared with either drug administered alone (Fig. 4).

#### Secondary pharmacodynamic outcome measures

**Figure 5:**
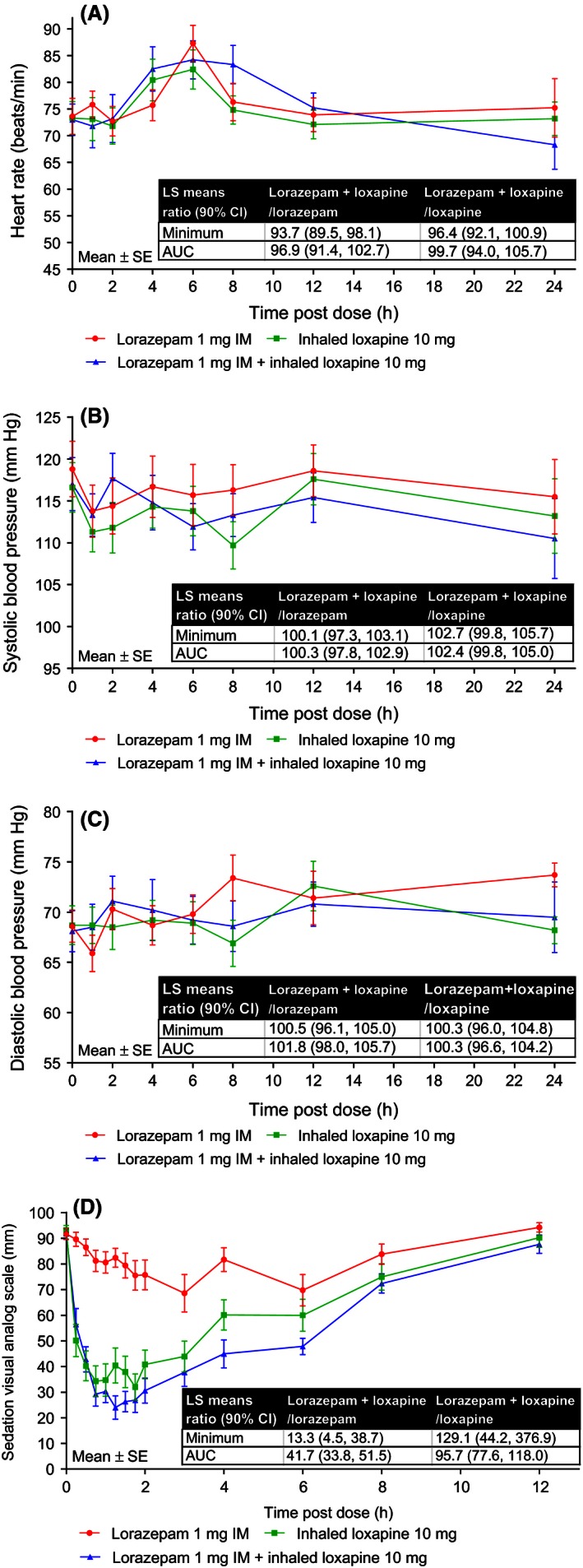
Secondary outcome measures. Heart rate (A); systolic blood pressure (B); diastolic blood pressure (C); sedation visual analog scale (D). AUC, area under the curve; CI, confidence interval; IM, intramuscular; LS, least squares.

Sitting heart rate and systolic and diastolic blood pressure showed no difference for lorazepam 1 mg IM + inhaled loxapine 10 mg compared with either drug administered alone (Fig. 5A–C). There was a greater effect on the VAS minimum value (mean [SD] 18.7 [16.2]) and AUC (mean [SD] 75.6 [33.1]) observed for lorazepam 1 mg IM + inhaled loxapine 10 mg combined treatment, compared with both inhaled loxapine 10 mg (minimum value mean [SD] 25.0 [19.6], AUC 84.7 [43.3]) and lorazepam 1 mg IM (minimum value mean [SD] 69.3 [26.3], AUC 164 [30.5]) treatments alone (Fig. 5D). However, while the LS mean ratios indicated there was a difference between lorazepam 1 mg IM and lorazepam 1 mg IM + inhaled loxapine 10 mg in AUC values for sedation (LS mean ratio 41.7 [90% CI 33.8, 51.5]), there was no difference between the combined treatment and inhaled loxapine 10 mg (LS mean ratio 95.7 [90% CI 77.6, 118]) (Fig. 5D).

Results from the CogScreen subtests (not shown) showed sedative effects for all three treatments on information processing speed, reaction time, speed variability, and psychomotor coordination. In these healthy volunteers, effects on these measures were statistically significantly greater with lorazepam 1 mg IM + inhaled loxapine 10 mg combined treatment than with either drug alone, particularly on the cognitive and psycho motor variables. The effects were evident 30 min postdose and reached their peak 1 h post dose for most measures, decreasing over the next 7 h.

### Safety

Concomitant administration of lorazepam 1 mg IM+ inhaled loxapine 10 mg and administration of each drug alone were well tolerated. Three subjects experienced treatment‐emergent AEs (Table 2): 1 during the open‐label part of the study (nasal congestion and palpitations) and 2 during the double‐blind part of the study (myalgia and headache). All of these AEs were considered mild and unrelated to the treatment. There were no dropouts, and no deaths, severe AEs, or premature discontinuations due to AEs were reported. No clinically important hematology, blood chemistry, urinalysis, vital sign, physical examination, or ECG results were observed.

**Table 2:** Safety summary

	Open‐label part (*N* = 4)	Crossover part (*N* = 18)	Overall (*N* = 22)
Number of patients experiencing an AE, *n* (%)	1 (25)	2 (11.1)	3 (13.6)
Total number of AEs, *n*	2	2	4
Palpitations	1	0	1
Nasal congestion	1	0	1
Myalgia	0	1	1
Headache	0	1	1
Total number of treatment‐related AEs, *n*	0	0	0

AE, adverse event.

## Discussion

This study investigated the tolerability and PD of concomitant administration of inhaled loxapine with IM lorazepam, the most common benzodiazepine used in combination with other drugs for the treatment of agitation. The results showed that concomitant administration of inhaled loxapine and lorazepam in healthy volunteers showed no difference in its effect on respiration rate or pulse oximetry (the primary endpoints) versus either drug alone, suggesting that the combined treatment is no more likely to result in respiratory depression than either drug administered alone. Evaluation of the secondary PD endpoints showed increased sedation effects (lower mean minimum value and AUC), using a subject self‐reported VAS‐based measure, for the combined treatment and for inhaled loxapine treatment alone compared with IM lorazepam in these healthy volunteers, although there was no difference in sedation between the combined treatment and inhaled loxapine alone on sedation over the duration of the study. The VAS sedative effects of lorazepam 1 mg IM + inhaled loxapine 10 mg appeared to be additive when administered in combination, similar to other combined antipsychotic/benzodiazepine treatments (Battaglia et al. 1997; Bieniek et al. 1998).

The CogScreen subtest results (not presented here) are consistent with the VAS assessment and demonstrate CNS sedative effects for IM lorazepam and inhaled loxapine administered alone and in combination. Taken together, these data suggest that care is required when inhaled loxapine is co‐administered with other CNS drugs, consistent with the usage of other antipsychotics. For example, concomitant administration of haloperidol with lorazepam, a common combination used to treat agitation, can lead to oversedation (Battaglia et al. 1997; Bieniek et al. 1998). Existing literature on co‐administration of oral loxapine with other CNS depressants (e.g., benzodiazepines, tricyclic antidepressants, general anesthetics, phenothiazines, sedatives/hypnotics, muscle relaxants, and/or illicit CNS depressants) recommends caution because of the potential increase in the risk of respiratory depression or respiratory failure, hypotension, profound sedation, and syncope (Heel et al. 1978; DePaulo and Ayd 1982; Battaglia et al. 1989). Furthermore, if benzodiazepine administration is necessary in addition to loxapine, it is recommended that subjects be monitored for excessive sedation and for orthostatic hypotension (Battaglia et al. 1989; Ereshefsky 1999; EMA 2013b).

No respiratory AEs were reported in this study, which included nonobese healthy volunteers who were current, ex‐ or non‐smokers but excluded those with reactive airway disease (Gross et al. 2014). This study showed that the inhaled loxapine‐lorazepam combined administration is well tolerated, with only three treatment‐emergent AEs. The AEs reported here were considered mild and unrelated to the study drug.

Several limitations should be acknowledged. Although falling within the recommend 1–2 mg dose level for concomitant administration (Wilson et al. 2012) in standard practice, the 1‐mg IM lorazepam dose used in this study was lower than that used in some previous studies assessing the effects of concomitant administration of antipsychotics with up to 2‐mg lorazepam for the treatment of agitation (Battaglia et al. 1997; Bieniek et al. 1998; Currier and Simpson 2001). Another possible limitation of this study could be the small sample size used; however, the sample size of the double‐blind part of the study and the randomization in a three‐way Williams square crossover design are consistent with the convention for studies conducted to examine PD profiles (FDA 2013) and did demonstrate no effect (equivalence) for the primary outcome measures. Inhaled loxapine is currently approved in the European Union for a maximum of two consecutive doses, administered 2 h apart (EMA 2013b). The tolerability and PD of concomitant administration of inhaled loxapine with IM lorazepam were not assessed under these conditions in this study and may therefore differ from the results seen here. Finally, the healthy volunteers assessed in this study may not be representative of the population being treated, who may have other comorbid conditions, may be taking other medications that might affect the overall results, or may have different sensitivities to the sedative effects of either drug alone or in combination.

In conclusion, the results of this study demonstrate the tolerability of inhaled loxapine administered concomitantly with lorazepam. No differences in respiration PDs or vital signs were seen when inhaled loxapine was administered in combination with IM lorazepam when compared with each drug alone, although there was a significant effect on sedation for the combined treatment in this population of healthy volunteers. The CogScreen subtest results showed that all treatments have CNS sedative effects, but these were greater with the combined treatment on the cognitive and psychomotor variables, suggesting that higher doses of lorazepam combined with inhaled loxapine could lead to greater cognitive impairment. Co‐administration of lorazepam 1 mg IM with inhaled loxapine 10 mg was well tolerated in this healthy population with no serious AEs reported.

## Author Contributions

*Participated in research design:* Spyker, Cassella.

*Conducted experiments:* Cassella, Stoltz.

*Performed data analysis:* Spyker, Cassella, Yeung.

*Wrote or contributed to the writing of the manuscript:* Spyker, Cassella, Stoltz, Yeung.

## Disclosures

J. V. C. is an employee of Alexza Pharmaceuticals, Inc. D. A. S. is a consultant employed by Alexza Pharmaceuticals, Inc. R. R. S. is an employee of Covance Clinical Research Unit. P. P. Y. is an employee of Teva Pharmaceuticals.
